# Nebulised pentamidine prophylaxis of *pneumocystis* pneumonia in adults accessing HIV services at royal free hospital, London

**DOI:** 10.1177/09564624241245155

**Published:** 2024-04-12

**Authors:** Malin Bergstrom, Anika Rahim, Jane Akodu, Gavin Marshall, Cora Harrison, Louisa Penrose, Marc CI Lipman, Robert F Miller

**Affiliations:** 1 HIV Services, Royal Free London NHS Foundation Trust, 158987 Royal Free Hospital, London, UK; 2 Pharmacy Services, Royal Free London NHS Foundation Trust, 158987 Royal Free Hospital, London, UK; 3 Medicine and Urgent Care, Royal Free London NHS Foundation Trust, 158987 Royal Free Hospital, London, UK; 4 UCL Respiratory, Division of Medicine, 4919 University College London, London, UK; 5 Respiratory Medicine, Royal Free London NHS Foundation Trust, 158987 Royal Free Hospital, London, UK; 6 Centre for Clinical Research in Infection and Sexual Health, Institute for Global Health, 4919 University College London, London, UK

**Keywords:** Nebulised pentamidine, prophylaxis, Pneumocystis, co-trimoxazole, adverse drug reaction

## Abstract

Receipt of nebulised pentamidine in people with HIV was audited to identify if individuals were appropriately receiving nebulised pentamidine, and whether national guidelines were being followed when prophylaxis was commenced and discontinued. Of 76 people with who received nebulised pentamidine, the main indication for starting nebulised pentamidine was a co-trimoxazole adverse drug reaction. Co-trimoxazole desensitization was not attempted before starting nebulised pentamidine. The main indication for stopping nebulised pentamidine prophylaxis was when immune reconstitution occurred. This single centre audit revealed that national guidelines were being followed in most cases. The lack of information regarding the reason for starting or stopping nebulised pentamidine prophylaxis, or detail of the clinician’s concerns about potential poor adherence with oral regimens of prophylaxis as a reason for choosing nebulised pentamidine prophylaxis, identifies a need for improved documentation of clinicians’ decision-making. Introduction of pharmacist-led interventions/alerts using patients’ electronic records, similar to those used in primary care, would enable the specialist pharmacy team to identify when and if co-trimoxazole desensitization has been offered and discussed/declined before a clinician prescribes nebulised pentamidine as well as enabling identification of those in who pentamidine prophylaxis has been continued, despite “immune reconstitution”.

## Introduction

Co-trimoxazole is the drug of first choice for both primary and secondary prophylaxis of *Pneumocystis* *jirovecii* pneumonia in people with HIV.^1,2^ Rash, with or without fever, and other adverse events occur in up to 20% of people. Recommended alternative prophylaxis regimens for those unable to tolerate co-trimoxazole include dapsone (given alone or combined with pyrimethamine and folinic acid), atovaquone, and nebulised pentamidine.^1,2^ Individuals who have glucose-6-phosphate dehydrogenase (G6PD) enzyme deficiency should not receive co-trimoxazole, or dapsone.^1,2^ Nebulised pentamidine is better tolerated, but less effective, and significantly more expensive than co-trimoxazole, or dapsone given alone or with pyrimethamine/folinic acid, for both primary and secondary prophylaxis of *Pneumocystis* pneumonia.^1–7^ Prophylaxis can be discontinued in people who immune reconstitute to CD4 ≥200 cells/ μ L, or to CD4 ≥100 cells/ μ L with an undetectable HIV viral load for ≥3 months.^1,2,8,9^

We retrospectively audited use of nebulised pentamidine prophylaxis in people accessing HIV services at Royal Free Hospital London (RFH) to identify if patients were appropriately receiving nebulised pentamidine prophylaxis, and whether national guidelines^2^ were being followed when NPP was commenced and when it was discontinued.

## Methods

The RFH is a large central London teaching hospital with an HIV referral centre that manages a large cohort of people with HIV (approximately 3200, in June 2022).

Adults (≥18 years) who received nebulised pentamidine prophylaxis on ≥1 occasion between 01 January 2017 and 31 December 2022 were included in this audit. Pentamidine (300 mg) was administered monthly, using a Respironics InnoSpire Deluxe (Phillips, Farnborough, UK) nebuliser.

Data were obtained from the RFH Pharmacy database, HIV services (Ian Charleson Day Centre [ICDC]) database & the hospital Electronic Document and Records Management (EDRM) system.

We recorded the reason why each person was receiving nebulised pentamidine prophylaxis (primary or secondary prophylaxis), whether nebulised pentamidine prophylaxis was commenced because of adverse events from co-trimoxazole (cutaneous reaction, neutropenia, biochemical hepatitis, drug fever), intolerance of co-trimoxazole (nausea, vomiting, diarrhoea, abdominal pain, anorexia), confirmed G6PD deficiency, or a clinician’s decision based on concerns that an individual might have adherence issues if receiving a daily prophylaxis regimen, and CD4 count (and %), and HIV viral load when nebulised pentamidine was started.

Additionally, we recorded why nebulised pentamidine prophylaxis, once commenced, was later stopped (CD4 count incremented to ≥200 cells/ μ L after starting antiretroviral therapy, CD4 ≥100 cells/ μ L with an undetectable viral load on ART, patient did not attend for nebulised pentamidine or lost to follow up, a clinician’s decision to stop nebulised pentamidine, or other reason) or was continued (failure of the CD4 count to increment after starting antiretroviral therapy, failure to stop after immune reconstitution to CD4 ≥200 cells/ μ L or after CD4 ≥100 cells/ μ L with an undetectable HIV, ART not started, a clinician’s decision to continue). We also recorded if a person had an episode of *Pneumocystis* pneumonia while receiving nebulised pentamidine prophylaxis.

The audit was registered as a clinical audit project with Royal Free London NHS Foundation Trust (Royal Free Hospital site) in January 2023.

## Results

From the RFH Pharmacy records we identified that 561 people received PCP prophylaxis between 01 January 2017 and 31 December 2022. Of these, 411 received co-trimoxazole, 23 received dapsone and 47 received atovaquone. Eighty people were prescribed nebulised pentamidine on ≥1 occasion over the 5 year period. Four people were excluded; in 3 nebulised pentamidine prophylaxis was administered for non-HIV associated reasons (2 post renal transplant, during chemotherapy for a neuroendocrine tumour in 1: all 3 had undetectable viral loads and CD4 counts >200 cells/ μ L): one further patient had HIV-2. Thus, 76 people are included in this audit. Their median age was 52 years (range 20 – 77); 52 were male. Sixty-six received primary and 10 received secondary prophylaxis. The main indication for starting nebulised pentamidine prophylaxis was a co-trimoxazole adverse drug reaction. The main reasons for discontinuing nebulised pentamidine prophylaxis was when immune reconstitution to CD4 ≥200 cells/ μ L occurred, or to CD4 ≥100 cells/ μ L with an undetectable HIV viral load. Reasons for starting, stopping or continuing nebulised pentamidine prophylaxis are shown in Table 1. Desensitization had not been attempted in any of those who had experienced an adverse reaction to co-trimoxazole before nebulised pentamidine prophylaxis was started. No-one developed *Pneumocystis* pneumonia while receiving nebulised pentamidine prophylaxis.

**Table 1.** Reasons for starting, stopping or continuing nebulised pentamidine prophylaxis, and laboratory results in 76 people with HIV.

Variable	Primary prophylaxis *n* = 66	Secondary prophylaxis *n* = 10
Reason for starting nebulised pentamidine:		
ADR with co-trimoxazole^a^	31	9
Intolerant of co-trimoxazole	2	0
G6PD deficiency	8	0
Clinician’s concern re adherence with oral		
prophylaxis	8	1
Other reason	10^b^	0
Not documented	7	0
When nebulised pentamidine started:		
CD4 count, cells/ μ L, (median (IQR)	72 (38 – 168)	83 (29 – 212)
CD4 %, median (IQR)	7 (3 – 11)	8 (2 – 18)
On ART	50	8
Viral load undetectable*	16	4
Number of doses of nebulised pentamidine, range, median	1 – 37 (6)	2 – 27 (5)
Reason for stopping nebulised pentamidine		
CD4 ≥200 cells/ μ L on ART for ≥3 months	24	3
CD4 ≥100 cells/ μ L, undetectable* viral load on ART	10	0
Patient DNA appointments for nebulised pentamidine/LTFU	9	2
Clinical decision	7	0
Other reason	8^c^	2^d^
Not recorded	5	1
When nebulised pentamidine stopped:		
CD4 count, cells/ μ L, median (IQR)	196 (101 – 110)	195 (177 – 212)
CD4 %, median (IQR)	11 (7 – 18)	11 (3 – 16)
On ART	56	7
Viral load undetectable*	43	4
Reason for not stopping nebulised pentamidine:		
CD4 count did not increment to ≥200 cells/ μ L with ART	2	1
Failure to stop prophylaxis despite CD4 ≥200 cells/ μ L with ART	1	0
Failure to stop prophylaxis despite CD4 ≥100 cells/ μ L& undetectable* viral load for 3 months with ART	0	1

Key: ADR = adverse drug reaction; G6PD = glucose-6-phosphate dehydrogenase; ART = antiretroviral therapy; DNA = did not attend; LTFU = lost to follow up; *<40 copies/mL; IQR = inter-quartile range.

^a^rash = 16 (including Drug Reaction with Eosinophilia and Systemic Symptoms = 2), haematological toxicity = 7, renal toxicity = 5, biochemical hepatitis = 3, anaphylaxis = 2, other reason = 4 (hypoglycaemia, itching, palpitations, hallucinations = 1 each), not specified = 4.

^b^pre-existing renal disease = 2, pre-existing neutropenia = 2, severe anaemia = 1, to avoid additive toxicity between co-trimoxazole and (tacrolimus = 1, methotrexate = 1), immune thrombocytopenic purpura = 1, pregnancy = 1, awaiting percutaneous gastrostomy (PEG) insertion (unable to swallow) = 1.

^c^shielding during COVID = 5, patient moved abroad = 1, PEG inserted = 1, on clinical review no evidence of ADR to co-trimoxazole = 1.

^d^wanted to change to co-trimoxazole (previously intolerant) = 1, declined further nebulised pentamidine = 1.

## Discussion

This audit identified that national guidelines for use of nebulised pentamidine prophylaxis were being followed in a majority of cases. However, lack of information regarding the reason for starting or stopping nebulised pentamidine prophylaxis, in 9% and 8% respectively, or specific detail of the concerns expressed by clinicians regarding potential poor adherence with oral regimens of prophylaxis as a reason for choosing nebulised pentamidine prophylaxis, identifies a need for improved documentation of clinicians’ decision-making.

British HIV Association Opportunistic Infection Guidelines suggest that clinicians might consider co-trimoxazole desensitization for people living with HIV who experience a non-severe (grade 3, or less) co-trimoxazole-associated reaction while receiving pneumocystis prophylaxis, but that desensitization should not be attempted in those who have experienced more severe reactions, e.g. drug rash with eosinophilia and systemic symptoms (DRESS), Stevens Johnson syndrome (SJS), or toxic epidermal necrolysis (TENS) [12]. In this audit it is of concern that, while our clinic has protocols in place for clinicians to order/carry out desensitization, none of those who had experienced (non-life threatening) adverse reactions to co-trimoxazole were offered desensitization before the clinician made the decision to commence nebulised pentamidine prophylaxis. Evidence-based recommendations support a clinician’s decision to discontinue prophylaxis when immune reconstitution occurs, following starting ART.^1,2,8,9^ Despite these recommendations, some people in this audit continued to receive nebulised pentamidine, despite evidence of “immune reconstituting”.

In primary care, greater involvement of pharmacists has resulted in safer prescribing in the longer term.^10^ Two information technology-based interventions have been used, computerised decision support (CDS) and pharmacist-led information technology intervention for medical errors (PINCER).^10,11^ CDS raises an alert when a clinician is about to prescribe medication that may potentially increase the risk of harm to an individual,^10^ PINCER searches individuals’ medical records to identify potential prescribing mistakes that have already happened.^10,11^

It is clear from this single centre audit that in terms of quality improvement, a prospective re-audit of use of nebulised pentamidine prophylaxis in our clinic after introduction of specialist pharmacist-led interventions/alerts using electronic patient records, similar to those already in use in primary care, would enable the specialist pharmacy team to identify when and if co-trimoxazole desensitization has been offered and discussed/declined before a clinician prescribes nebulised pentamidine^10,11^ as well as enabling identification of those in who pentamidine prophylaxis has been continued by a clinician, despite “immune reconstitution”.

As this was a single centre audit and had small numbers, its findings might not be generalisable to other treatment centres. A multi-centre or national audit would provide a better picture of current use of nebulised pentamidine prophylaxis in people with HIV in UK.
